# Respiratory Syncytial Virus Seasonality, Beijing, China, 2007–2015

**DOI:** 10.3201/eid2506.180532

**Published:** 2019-06

**Authors:** Jianxing Yu, Chunyan Liu, Yan Xiao, Zichun Xiang, Hongli Zhou, Lan Chen, Kunling Shen, Zhengde Xie, Lili Ren, Jianwei Wang

**Affiliations:** National Health Commission Key Laboratory of Systems Biology of Pathogens, Beijing, China (J. Yu, Y. Xiao, Z. Xiang, H. Zhou, L. Chen, L. Ren, J. Wang);; Institute of Pathogen Biology of Chinese Academy of Medical Sciences and Peking Union Medical College, Beijing (J. Yu, Y. Xiao, Z. Xiang, H. Zhou, L. Chen, L. Ren, J. Wang);; Capital Medical University Beijing Children’s Hospital, Beijing (C. Liu, K. Shen, Z. Xie)

**Keywords:** respiratory syncytial virus, temporal trends, seasonality, epidemiology, etiology, viruses, China, respiratory infections, pneumonia, children, pediatric population, season, RSV-A, RSV-B

## Abstract

During July 2007–June 2015, we enrolled 4,225 hospitalized children with pneumonia in a study to determine the seasonality of respiratory syncytial virus (RSV) infection in Beijing, China. We defined season as the period during which >10% of total PCRs performed each week were RSV positive. We identified 8 distinctive RSV seasons. On average, the season onset occurred at week 41 (mid-October) and lasted 33 weeks, through week 20 of the next year (mid-May); 97% of all RSV-positive cases occurred during the season. RSV seasons occurred 3–5 weeks earlier and lasted ≈6 weeks longer in RSV subgroup A–dominant years than in RSV subgroup B–dominant years. Our analysis indicates that monitoring such RSV subgroup shifts might provide better estimates for the onset of RSV transmission. PCR-based tests could be a flexible or complementary way of determining RSV seasonality in locations where RSV surveillance is less well-established, such as local hospitals throughout China.

Respiratory syncytial virus (RSV) is a major cause of lower respiratory tract infection in young children worldwide (*1*–*3*); 2.7–3.8 million hospitalizations and 94,600–149,400 deaths occur each year among children <5 years of age as a result of RSV infection (*4*). Studies have also demonstrated the contribution of RSV to respiratory tract infections in adults (*5*,*6*). However, no licensed RSV vaccine is available (*7*), and the only approved specific therapy, palivizumab (anti-RSV antibody), has limited uses among infants at high risk for severe respiratory illness in high-resource settings (*8*).

RSV causes epidemics in the winter in regions with temperate climates (*8*,*9*). However, spatiotemporal variations have been observed in the timing of RSV activity (*10*,*11*), and knowledge of the exact timing is helpful for guiding healthcare providers and health officials on the timing of diagnostic testing and immunoprophylaxis for infants at high risk for infection (*12*). RSV circulation is monitored in the United States year-round through the National Respiratory and Enteric Virus Surveillance System (*10*) and in 15 countries of Europe through the European Influenza Surveillance Network (*13*). Since 2017, the World Health Organization has also conducted RSV surveillance to guide its global RSV prevention strategy (*9*) using the Global Influenza Surveillance and Response System (*14*). However, most of the real-time data on RSV seasonality comes from RSV surveillance, and data are lacking in many places of the world. Because disease surveillance is labor- and resource-intensive, information on seasonality from other sources is needed.

China has a high burden of RSV infection (*4*), but RSV surveillance in this country is less established, and implementation of diagnostic tests is limited. Several previous studies reported an RSV prevalence of 17%–33% among children with severe acute respiratory illness (*15*,*16*), but few have assessed the seasonality or trends of RSV infections in China. Not having data available on RSV seasonality in China could encumber implementation of therapy and prophylactic interventions for RSV.

Since July 2007, we have been monitoring for RSV infection among hospitalized children with pneumonia in Beijing, China. In this study, we evaluated the PCR results collected in 8 consecutive years (2007–2015) to characterize the seasonality of RSV by year. Also, because disease and death attributable to RSV varies from year to year (*4*,*11*,*17*), we explored the factors that might affect RSV activity.

## Materials and Methods

### Study Design

During July 1, 2007–June 30, 2015, we enrolled children with pneumonia who were admitted into wards of the Departments of Respiratory Medicine, Infectious Diseases, and Emergency Medicine and the pediatric intensive care unit (PICU) of Beijing Children’s Hospital (Beijing, China). We recruited children 28 days–13 years of age who had symptoms of acute infection, defined as fever (body temperature ≥38.0°C) or hypothermia (body temperature <35.5°C), leukocytosis (leukocyte count >15,000 cells/mL for children <5 years of age or >11,000 cells/mL for children ≥5 years of age), or leukopenia (leukocyte count <5,000 cells/mL for children <5 years of age or <4,000 cells/mL for children ≥5 years of age); had >1 respiratory sign or symptom (i.e., cough, sputum production, shortness of breath, tachypnea [>60 breaths/min for patients <2 months of age, >50 breaths/min for patients 2–11 months of age, >40 breaths/min for patients 12–59 months of age, and >30 breaths/min for patients >5 years of age], wheezing or crackles, dyspnea, or chest pain); and had radiographic evidence suggestive of pneumonia (e.g., chest radiograph showing consolidation, infiltrates, or pleural effusion). We excluded children known to be immunosuppressed (defined as having received a solid organ or hematopoietic stem cell transplant, undergoing chemotherapy, having a history of HIV or AIDS, or using steroids for >30 days).

We obtained informed consent from each child’s parents or guardians before enrollment. The study protocol was approved by the ethics review committee at the Institute of Pathogen Biology, Chinese Academy of Medical Sciences, Beijing, China.

### Specimen Collection and Laboratory Testing

We transferred the nasopharyngeal aspirates of each enrolled patient into Universal Transport Medium (Copan Group, https://www.copangroup.com), distributed them into aliquots, and stored them at −80°C. We screened for RSV subgroup A (RSV-A) and B (RSV-B) and other common respiratory viruses, including influenza virus (A and B), human rhinovirus, human parainfluenza viruses 1–4, human adenovirus, human enterovirus, human bocavirus, human metapneumovirus, and human coronavirus (229E, OC43, NL63, and HKU1), using multiplex reverse transcription PCR and PCR assays as described (*18*).

### Data Collection

At enrollment, using a standardized reporting form, we collected demographic data (sex and age), epidemiologic data (date of illness onset and history of prematurity [defined as birth at gestational age <37 weeks]), and clinical data (signs, symptoms, and concurrent medical conditions). Concurrent medical conditions included congenital heart disease (CHD; i.e., children with an International Classification of Diseases, Ninth Revision [ICD-9], diagnostic code 745.xx, 746.xx, or 747.xx), chronic lung disease (e.g., bronchopulmonary dysplasia), chromosomal anomalies (e.g., Down syndrome), moderate to severe anemia (hemoglobin <90 g/L), and malnutrition. We also collected data on clinical outcomes, including PICU admission, noninvasive ventilation (e.g., continuous positive airway pressure), invasive ventilation (i.e., mechanical ventilation involving tracheostomy or endotracheal tube), acute respiratory failure (ICD-9 code 518.81), shock (ICD-9 code 785.5x), sepsis (ICD-9 code 038.xx), and death, by abstracting data from medical charts.

### Statistical Analysis

We fitted a logistic regression model (with sine and cosine functions of the illness onset week) to individual patient data and retained a seasonal curve as described previously (*19*). To profile the seasonality of RSV with a smooth seasonal curve, we adopted an approach described previously by the US Centers for Disease Control and Prevention: we defined the RSV season as consecutive weeks during which the percentage of tests positive for RSV per week exceeded a threshold of 10% (*20*,*21*). The peak was expected to occur between the dates of onset and offset, unless an outbreak took place outside of the season.

We used a multivariable logistic regression model to explore the factors that could affect RSV transmission over a period of years. We introduced a pair of sine and cosine functions of illness onset week into the model as described in the previous paragraph. We modeled age and year using a restricted cubic spline with 5 knots (*22*). We also introduced into the model other factors that differed significantly (p<0.10 in bivariate analysis), such as sex, dominant RSV subgroup in each season, concurrent medical conditions, and PICU admission during hospitalization. We conducted all analyses in R version 2.15.3 (https://cran.r-project.org) using mgcv package version 1.8–15 (*23*).

## Results

### Characteristics of Patients

**Figure 1:**
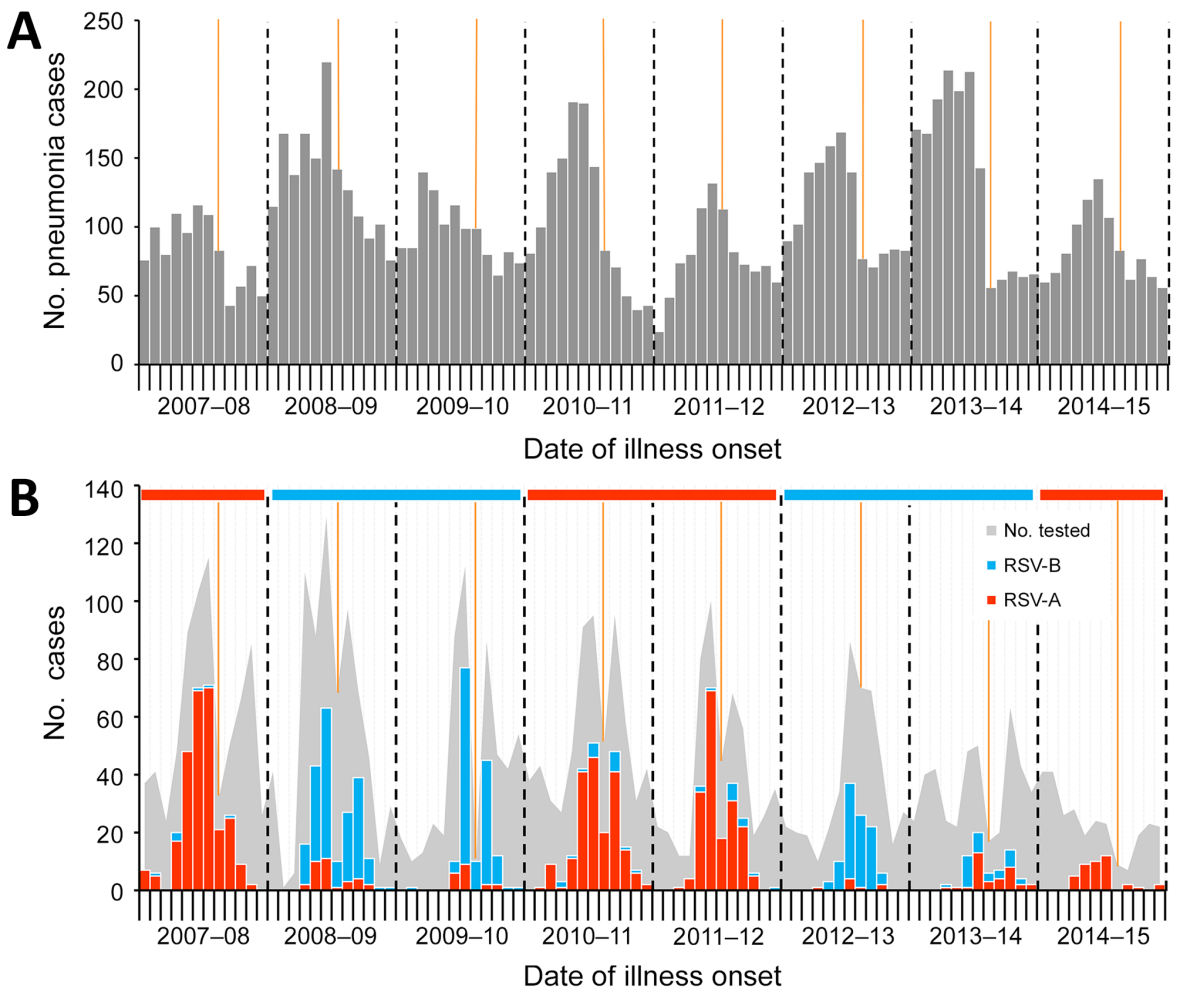
Cases of pneumonia and RSV infection in hospitalized children 28 days–13 years of age, by month, Beijing, China, July 1, 2007–June 30, 2015. A) Cases of pneumonia (defined as International Classification of Diseases, 9th Revision, codes 480–488), including children not enrolled in the study (n = 9,950). B) RSV-positive cases (n = 1,270), by subgroup. The shaded area represents the total number of children enrolled in study (n = 4,225). The horizontal ribbon on top of the chart denotes the dominant RSV subgroup for that year. We assigned 2013–14 as an RSV-B–dominant season for the purposes of modeling, although the numbers of RSV-A and RSV-B cases were almost equal. Orange vertical lines denote the Chinese Spring Festival; dashed vertical lines indicate divisions between seasons. RSV, respiratory syncytial virus; RSV-A, RSV subgroup A; RSV-B, RSV subgroup B.

During the study period, 9,950 children with a primary diagnosis of pneumonia (ICD-9 codes 480–488) were admitted to Beijing Children’s Hospital (Figure 1, panel A). Of these, 4,225 patients (2,604 [62%] boys, 1,621 [38%] girls) were recruited into the study (Table 1). Median age was 1.4 (interquartile range 0.4–6.1) years. Of the recruited patients, 493 (12%) had a concurrent medical condition at the time of hospital admission. The most frequent condition was CHD (7%, 278/4,225), followed by history of prematurity (4%, 182/4,225), chronic lung disease (1%, 28/4,225), anemia (<1%, 21/4,225), malnutrition (<1%, 13/4,225), and chromosomal anomalies (<1%, 3/4,225). During the 8-year study, the number enrolled decreased from 718 in the 2007–08 RSV year to 282 in the 2014–15 RSV year, a 61% decrease. The age of children also differed each year (p<0.001); the last 2 years included more children >5 years of age.

**Table 1:** Demographic characteristics of hospitalized children with pneumonia, Beijing, China, July 1, 2007–June 30, 2015, by respiratory syncytial virus year*

Characteristics	2007–08	2008–09	2009–10	2010–11	2011–12	2012–13	2013–14	2014–15	p value
No. patients	718	693	522	650	495	438	427	282	
Sex									
M	455 (63)	418 (60)	363 (70)	387 (60)	309 (62)	260 (59)	251 (59)	161 (57)	0.003
F	263 (37)	275 (40)	159 (30)	263 (40)	186 (38)	178 (41)	176 (41)	121 (43)	
Age, y, median (IQR)	0.7 (0.2–3.8)	0.9 (0.3–3.3)	0.8 (0.2–2.7)	2 (0.6–6.1)	1.4 (0.4–6.1)	2.2 (0.5–7.3)	5 (2.1–8.6)	3.8 (0.6–7.5)	<0.001
Age group, mo†									<0.001
1–5	304 (42)	228 (33)	210 (40)	140 (22)	147 (30)	108 (25)	38 (9)	60 (21)	
6–11	127 (18)	154 (22)	89 (17)	84 (13)	68 (14)	60 (14)	28 (7)	28 (10)	
12–23	54 (8)	94 (14)	71 (14)	104 (16)	69 (14)	48 (11)	39 (9)	19 (7)	
24–59	78 (11)	82 (12)	61 (12)	118 (18)	60 (12)	63 (14)	110 (26)	55 (20)	
>60	155 (22)	135 (19)	91 (17)	204 (31)	151 (31)	159 (36)	212 (50)	120 (43)	
Underlying medical condition	68 (9)	103 (15)	65 (12)	88 (14)	71 (14)	44 (10)	32 (7)	22 (8)	<0.001
History of prematurity‡	8 (1)	23 (3)	24 (5)	36 (6)	29 (6)	27 (6)	22 (5)	13 (5)	<0.001
Congenital heart disease§	49 (7)	76 (11)	38 (7)	47 (7)	38 (8)	13 (3)	10 (2)	7 (2)	<0.001
Chronic lung diseases¶	6 (1)	5 (1)	2 (0)	6 (1)	4 (1)	3 (1)	0	2 (1)	0.700
Anemia#	5 (1)	2 (0)	5 (1)	4 (1)	1 (0)	3 (1)	0	1 (0)	0.424
Malnutrition	5 (1)	2 (0)	2 (0)	3 (0)	0	1 (0)	0	0	0.337
Chromosomal anomalies**	2 (0)	1 (0)	0	0	0	0	0	0	0.441

*Values are no. (%) except where indicated. IQR, interquartile range. †Some columns do not add up to 100% because of rounding. ‡Prematurity was defined as birth at gestational age <37 weeks. §Congenital heart disease (defined as International Classification of Diseases, Ninth Revision, codes 745.xx, 746.xx, or 747.xx) was diagnosed at hospital admission. ¶For example, bronchopulmonary dysplasia. #Moderate to severe anemia, defined as hemoglobin <90 g/L at hospital admission. **For example, Down syndrome.

**Figure 2:**
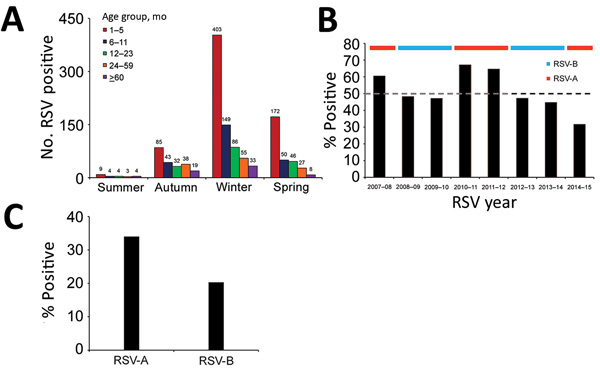
Hospitalized children with pneumonia testing positive for RSV, by age group, calendar season, and RSV subgroup, Beijing, China, July 1, 2007–June 30, 2015. A) Number of RSV-positive children (indicated by numbers above bars) by age group and season. Summer is defined as June–August, autumn as September–November, winter as December–February, and spring as March–May. B) Percentage of infants aged 28 days–5 months positive for RSV, by RSV season. The horizontal ribbon on top of the chart denotes the dominant RSV subgroup for that season. We assigned 2013–14 as an RSV-B–dominant season for the purposes of modeling, although the numbers of RSV-A and RSV-B cases were almost equal. Dashed line indicates 50% positivity. C) Percentage of infants 28 days–5 months of age positive for RSV, by RSV subgroup. RSV, respiratory syncytial virus; RSV-A, RSV subgroup A; RSV-B, RSV subgroup B.

Overall, RSV was identified in 1,270 (30%) children among the enrolled cohort. We observed a holiday effect for the Chinese Spring Festival (occurring in late January or early February) each year. A sharp decrease in sample number and samples positive for RSV were observed each year during this event, resulting in a bimodal distribution curve (Figure 1, panel B). Overall, 785 (62%) RSV-positive samples were RSV-A and 485 (38%) were RSV-B. Although almost equal numbers of RSV-A and RSV-B were identified in 2013–14, we assigned 2013–14 as an RSV-B–dominant season for modeling purposes. This assignment does not subvert our findings that subgroups A and B alternate biennially. Each RSV subgroup dominated for 2 consecutive years during our study period, for a total of 4 years each (RSV-A during 2007–08, 2010–11, 2011–12, and 2014–15; RSV-B during 2008–09, 2009–10, 2012–13, and 2013–14) (Figure 1, panel B). Of 1,270 RSV-positive children, 726 (57%) were ill during the winter (i.e., December–February), 669 (53%) were young infants 28 days–5 months of age (Figure 2, panel A), 113 (9%) had CHD, and 61 (5%) had a history of prematurity. Among infants 28 days–5 months of age with pneumonia, the average proportion positive for RSV was 54% (669/1,235); the proportion increased to 61%–67% in RSV-A–dominant years (2007–08, 2010–11, and 2011–12) but was <50% in RSV-B–dominant years (Figure 2, panel B).

During the study, 335 (8%) hospitalized children with pneumonia were admitted into the PICU, and 8 died (median age 1.4 years, range 4 months–13 years). RSV-positive children were more likely than RSV-negative children to be admitted into the PICU (positive 10% vs. negative 7%; p = 0.001), receive noninvasive ventilation (positive 20% vs. negative 10%; p<0.001), and have respiratory failure (positive 16% vs. negative 9%; p<0.001) (Table 2). Of the 8 deceased children, 2 were positive for adenovirus, 1 for enterovirus, and 1 for both human parainfluenza virus 1 and human bocavirus; none of the deceased children tested positive for RSV. All 8 deceased children were born at term, and only 2 had concurrent medical conditions at hospital admission (1 CHD, 1 IgA nephropathy).

**Table 2:** Clinical outcomes of children with pneumonia, by RSV positivity, Beijing, China, July 1, 2007–June 30, 2015*

Characteristic	All children, n = 4,225	RSV-positive children, n = 1,270	RSV-negative children, n = 2,955	p value
Admission into PICU	335 (7.9)	127 (10.0)	208 (7.0)	0.001
Mechanical ventilation	606 (14.3)	264 (20.8)	342 (11.6)	<0.001
Invasive	108 (2.6)	29 (2.3)	79 (2.7)	0.524
Tracheostomy	37 (0.9)	6 (0.5)	31 (1.0)	0.072
Endotracheal tube	99 (2.3)	25 (2.0)	74 (2.5)	0.319
Noninvasive†	553 (13.1)	258 (20.3)	295 (10.0)	<0.001
Respiratory failure	466 (11.0)	208 (16.4)	258 (8.7)	<0.001
Shock	21 (0.5)	2 (0.2)	19 (0.6)	0.053
Sepsis	94 (2.2)	21 (1.7)	73 (2.5)	0.112
Death	8 (0.2)	0	8 (0.3)	0.115

*All values are no. (%). PICU, pediatric intensive care unit; RSV, respiratory syncytial virus. †Includes continuous positive airway pressure.

### Trends of RSV Infection

**Figure 3:**
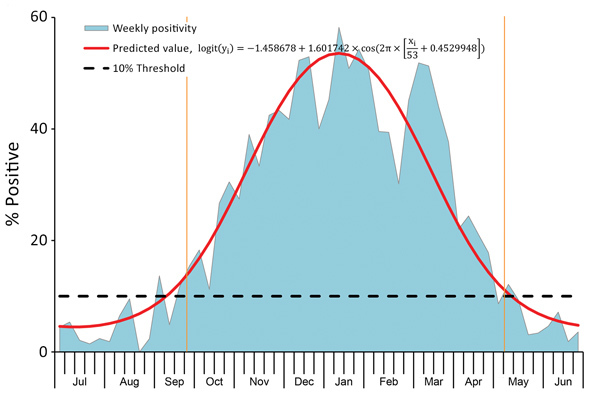
Average percentage of PCR tests positive for respiratory syncytial virus (RSV) per week among hospitalized children 28 days–13 years of age with pneumonia, Beijing, China, July 1, 2007–June 30, 2015. Graph begins at calendar week 27. A seasonal curve is superimposed onto the graph. RSV season was defined as consecutive weeks during which the percentage of RSV-specific PCRs testing positive per week exceeded a 10% threshold. Season onset and offset are indicated by the 2 orange vertical lines.

**Figure 4:**
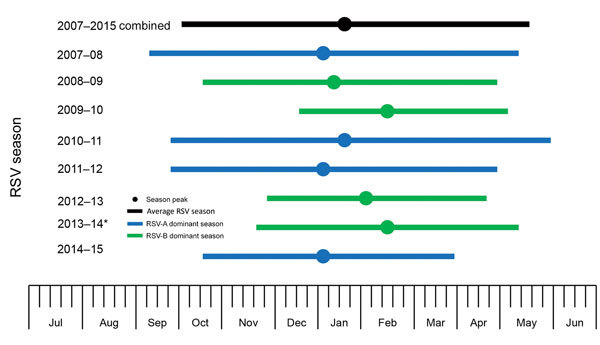
RSV season duration and peak, by year, Beijing, China, July 1, 2007–June 30, 2015. RSV season was defined as consecutive weeks during which the percentage of RSV-specific PCRs testing positive per week exceeded a 10% threshold. Graph begins at calendar week 27. *We assigned 2013–14 as an RSV-B–dominant season for modeling purposes, although almost equal numbers of RSV-A and RSV-B were identified that season. RSV, respiratory syncytial virus; RSV-A, RSV subgroup A; RSV-B, RSV subgroup B.

We identified 8 distinctive RSV seasons during the study period (July 1, 2007–June 30, 2015) using our model (p<0.001 for all), even though the number of RSV-positive children varied (from 41 to 280) by study year. The percentage of PCR tests positive for RSV each week throughout the summer months typically exceeded 3% but was <10%; once the 10% positivity threshold was exceeded, the percentage of tests positive for RSV increased rapidly. Overall, 97% of RSV-positive PCRs occurred within the period defined by the 10% threshold (Table 3; Figure 3). Using the 10% cutoff point and a fitted seasonal curve, we determined the following RSV season parameters for each of the 8 years of our study: season onset (first of 2 consecutive weeks during which RSV positivity in seasonal curve exceeded 10%), duration, peak (week with highest RSV positivity in seasonal curve), offset (last week that RSV positivity in seasonal curve exceeded 10%), and percentage of RSV-positive samples captured within the season. Data show that the average season onset occurred at calendar week 41 (mid-October) and lasted 33 weeks, through week 20 (mid-May) of the next year (Table 3). The peak of RSV activity occurred at calendar week 3 (mid-January). RSV circulated at low levels during off-seasons. Overall, 24 (3.3%) of 724 children tested were positive for RSV in summer, a finding consistent with Broberg et al. (*13*). Variations in RSV activity were observed from year to year (Figure 4). RSV activity in 2007–08, 2011–12, and 2014–15 (RSV-A–dominant years) peaked early (during calendar week 1) and in 2009–10 and 2013–14 (RSV-B–dominant years) peaked late (calendar week 7, in February). In general, the RSV season started earlier (RSV-A week 40 vs. RSV-B week 45), lasted longer (RSV-A 34 weeks vs. RSV-B 28 weeks), and peaked earlier (RSV-A week 2 vs. RSV-B week 5) in RSV-A–dominant seasons than in RSV-B–dominant seasons (Table 3).

**Table 3:** RSV positivity, by subgroup, and RSV season characteristics defined by the 10% positivity threshold, Beijing, China, July 1, 2007–June 30, 2015*

RSV year	No. RSV positive						Season duration, wk	% RSV-positive PCRs captured
Total	RSV-A:RSV-B ratio	Calendar week no. of season (date of last day of week)		
		Onset	Peak	Offset
2007–08	280	273:7		38 (2007 Sep 15)	1 (2008 Jan 5)	19 (2008 May 10)	35	95
2008–09	211	33:178		43 (2008 Nov 25)	3 (2009 Jan 17)	17 (2009 Apr 25)	28	92
2009–10	157	19:138		52 (2009 Dec 26)	7 (2010 Feb 13)	18 (2010 May 1)	20	93
2010–11	210	192:18		40 (2010 Oct 02)	3 (2011 Jan 15)	22 (2011 May 28)	36	94
2011–12	198	184:14		40 (2011 Oct 01)	1 (2012 Jan 7)	17 (2012 Apr 21)	31	98
2012–13	105	8:97		49 (2012 Dec 01)	5 (2013 Feb 2)	16 (2013 Apr 20)	21	94
2013–14†	68	35:33		48 (2013 Nov 30)	7 (2014 Feb 15)	19 (2014 May 10)	25	90
2014–15	41	41:0		43 (2014 Oct 25)	1 (2015 Jan 3)	13 (2015 Mar 28)	24	90
2007–15 combined‡	1,270	785:485		41 (mid-Oct)	3 (mid-Jan)	20 (mid-May)	33	97
RSV-A dominant§	729	690:39		40 (early Oct)	2 (early Jan)	20 (mid-May)	34	96
RSV-B dominant¶	541	95:446		45 (early Nov)	5 (early Feb)	19 (early May)	28	95

*RSV season was defined as consecutive weeks during which the percentage of RSV-specific PCRs testing positive per week exceeded a 10% threshold. RSV, respiratory syncytial virus; RSV-A, RSV subgroup A; RSV-B, RSV subgroup B. †We classified 2013–14 as an RSV-B–dominant season for the purpose of modeling, even though an almost equal number of RSV-A and RSV-B cases were identified in that season. ‡All children (n = 4,225) in the 8 years of the study (2007–2015) were included in a model to give an average estimate of RSV seasonal characteristics for 2007–15 combined. §Children who were ill during the 2007–08, 2010–11, 2011–12, and 2014–15 seasons (n = 2,145) were included in a model to give an average estimate of RSV seasonal characteristics for RSV-A–dominant seasons. ¶Children who were ill during the 2008–09, 2009–10, 2012–13, and 2013–14 seasons (n = 2,080) were included in a model to give an average estimate of RSV seasonal characteristics for RSV-B–dominant seasons.

### Factors Associated with RSV Infection

**Figure 5:**
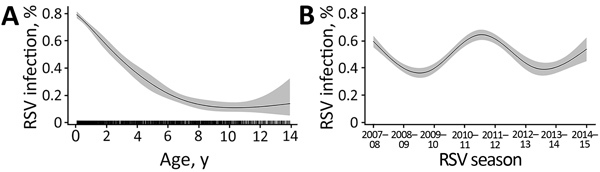
Analysis of nonlinear influence of age and RSV year on RSV activity among children 28 days–13 years of age hospitalized with RSV infection, Beijing, China, July 1, 2007–June 30, 2015. Graphs show effect of age (A) and RSV season (B) on probability of infection (p<0.001 for both). The rug plot along the *x* axis shows the observed values; gray shading indicates 95% CIs. RSV, respiratory syncytial virus.

We explored factors that significantly affected RSV activity by using multivariable modeling. After adjusting for week of illness onset, year, PICU admission, and CHD, age had a strong decreasing monotonic effect on RSV infection (p<0.001; Figure 5, panel A). We also observed a yearly cyclic pattern with a distinct periodicity of 4 years for RSV year in the response plot (p<0.001; Figure 5, panel B). However, when we introduced RSV-A–dominant year into the model as a factor to be adjusted for, the cyclic trends and the year’s association with RSV activity diminished (p = 0.11).

## Discussion

We performed a PCR-based RSV screening in a cohort of children with pneumonia in Beijing to assess RSV seasonality. Our findings show that on average the RSV season starts at calendar week 41 (mid-October) and lasts 33 weeks through week 20 of the next year (mid-May); 97% of total RSV-positive PCRs occur during this period. This seasonal pattern is highly consistent with that reported in the United States (*10*), a country at the same latitude as China in the Northern Hemisphere. The World Health Organization is expecting an RSV vaccine on the market within 5–10 years (*24*); ≈62 RSV vaccine candidates are under development, and 19 of them are undergoing clinical trials. Our study of RSV seasonality and trends in Beijing could inform vaccine development and the optimization of future vaccination strategies, such as the timing of administration (year-round or seasonal), target population (mothers or infants), and ingredients, for China, the country with the largest population in the world.

We used RSV percent positivity to determine RSV seasonality because this method is highly sensitive and can be used to define RSV season not only retrospectively at the end of the season but also during the year in real-world practice. The main disadvantage of this strategy is that results can be driven in large part by the denominator; the presence of other cocirculating pathogens that cause pneumonia, especially influenza viruses, can influence the parameters of the RSV season.

Although the number of PCRs performed each year varied considerably and a shift toward older age was evident among affected children in the last 2–3 years of the study, the seasonal pattern each year remained consistent and reproducible when classifying the study year by the prevailing RSV subgroup. During the 8 consecutive years of the study, the number of children admitted with pneumonia dropped from 718 in the first year to 282 in the last; RSV positivity also decreased remarkably, from 280 in the first year to 41 in the last. The reasons for this decrease might be attributable to a decrease in the occurrence of RSV-associated pneumonia in the pediatric population or, more likely, a fall in pediatrician interest for enrolling patients into the study over time, given we did not observe a simultaneously drastic drop of pneumonia cases in the hospital (Figure 1, panel A). Moreover, the demographic characteristics of patients also differed throughout the study years. In the first study year, more children 28 days–5 months of age (42%) were enrolled, and in the last, more children >5 years of age (43%) were enrolled. Despite this change, the observed seasonal characteristics in each year changed little if any, indicating that viral factors rather than demographic factors had more of an influence over RSV seasonality. When study years were classified by the prevailing RSV subgroup, the observed season onset, peak, and offset showed good reproducibility. We found that >90% of RSV-positive PCRs could be captured during the RSV season for all 8 study years, and the average capture was 97% for 2007–2015 combined. The method we used in our study (PCR-based testing) can be used by local hospitals in China where surveillance data are lacking to compile the extensive amount of data needed to assess RSV seasonality.

The shifting in dominance from RSV-A to RSV-B every 2 years was repeatedly observed in our study and others (*25*). These shifts have been shown to be strongly associated with changes in the dominant RSV strain circulating (*11*,*25*); however, this phenomenon has not been investigated in detail. Similar to previous studies (*8*,*26*,*27*), the positivity for RSV-A (19%, 785/4,225) was significantly higher than that for RSV-B (11%, 485/4,225; p<0.001) in our study. The season onset and peak in RSV-A–prevailing years occurred ≈3–5 weeks earlier and duration was ≈6 weeks longer than those observed in RSV-B–prevailing years. The response plot showing the effect of year on RSV activity also indicated patterns of regular peaks in the years RSV-A prevailed (i.e., 2007–08, 2010–11, 2011–12, and 2014–15), but the effect of the year diminished when RSV-A–prevailing year was adjusted for as a factor in the model, suggesting that repeated shifting between RSV-A and RSV-B might play a substantial role in driving RSV transmission dynamics in populations. This observation is also supported by a model proposed by White et al., who found that the transmission rate of RSV-A (8%) was slightly higher than that of RSV-B (*25*). Just like the influencing factors explored in other studies (e.g., geographic latitude and longitude, social and demographic factors, population density, and climate) (*11*,*13*,*22*), RSV subgroup replacement might play a key role in the activity of RSV. The alternating nature of RSV subgroups could explain the alternating early-big or late-small pattern and year-to-year variation in epidemic size and timing of RSV transmission observed previously (*11*,*25*). Our finding that RSV subgroup shifting was associated with RSV activity suggests that when using RSV seasonal data or conducting RSV surveillance, one should pay attention to the prevailing subgroups in the season to optimize the timing of immunoprophylaxis. This finding indicates the significance of genotyping in RSV surveillance.

Although RSV-A cases outnumbered RSV-B in our study, we did not determine the reason for this finding, whether RSV-A caused more symptomatic illness or transmitted more quickly than RSV-B among children. The relationship between RSV subgroup infection and disease severity is still controversial (*8*,*26*,*28*,*29*), and this issue warrants further study.

In our study, more than half of the RSV hospitalizations occurred in infants 28 days–5 months of age. A monotonic decreasing effect on the activity of RSV with age was observed in the response plot (Figure 5, panel A), indicating that younger age is a risk factor for RSV infection. Children who were in their first months of life had the highest risk for RSV infection (*3*,*30*). Administration of 1 dose of palivizumab (15 mg per kg body weight) each month for 5 months has been recommended to protect children at high risk for severe respiratory infection (e.g., preterm infants and infants with CHD in their first year of life) (*12*). As of March 2019, palivizumab is not licensed for use in China, and no immunoprophylaxis is available to prevent severe RSV infection. One third (30%) of children in our study with pneumonia requiring hospitalization had RSV infection; this finding is similar to previous estimates of 28%–34% in other countries (*1*,*2*). Considering the high positivity rate of RSV in children with pneumonia, RSV-associated illness should be considered a high priority for public health authorities in China. Our study of the relationship between age and RSV infection gives urgency to developing RSV diagnostics and indicates the need to study long-lasting and high-affinity new therapeutics and vaccines in the future (*9*,*31*).

Our study has some limitations. First, our study was conducted at 1 local hospital. Because no national RSV seasonality data were available for comparison, whether our results could represent other regions in China with an RSV burden is unknown. However, overall, our data are comparable with those observed in the United States. In addition, a previous study conducted in 15 countries of Europe showed that RSV seasons peaked later and lasted longer with increasing latitude (*13*). Because China is a large country that spans several geographic zones and climates, further studies are needed at other locations to fully characterize RSV seasonality in China. Second, our study was conducted in children with pneumonia, a population that usually has a high RSV positivity rate. Seasonality should also be evaluated in children with mild symptoms and in adults. Third, we did not evaluate the size of the local hospital or the minimum number of tests needed each year to conduct a more reliable analysis of RSV seasonality. Fourth, we excluded children without fever from our study. Because illness caused by RSV can manifest without fever, particularly among infants (*32*), the case definition used in our study was suboptimal, and we might have missed some children infected with RSV. Considering no international RSV case definition exists (*13*), we encourage researchers in future studies to determine a working case definition that can balance the many attributes (e.g., accuracy, feasibility, flexibility, and usefulness) of the various definitions that have been used to conduct RSV surveillance or study disease burden.

In conclusion, RSV infection showed distinctive seasonal patterns in Beijing, China. The prevailing RSV subgroup in a given season appears to affect the timing of RSV activity. Monitoring alterations of RSV subgroups might provide a better and more comprehensive description of RSV transmission and trends. A PCR-based diagnostic test at local hospitals could be a useful tool to determine RSV seasonality in circumstances where RSV season is unknown or surveillance is less established.
